# Antibody lineages with evidence of somatic hypermutation persisting for >4 years in a South African subject with broad neutralizing activity

**DOI:** 10.1186/1742-4690-9-S2-P85

**Published:** 2012-09-13

**Authors:** M Moody, AM Trama, M Bonsignori, C Tsao, MS Drinker, TC Gurley, JD Amos, JA Eudailey, LC Armand, R Parks, KE Lloyd, S Wang, K Seo, J Lee, KJ Jackson, R Hoh, T Pham, KM Roskin, SD Boyd, AZ Fire, ES Gray, L Morris, H Liao, GD Tomaras, TB Kepler, G Kelsoe, BF Haynes

**Affiliations:** 1 Duke University Medical Center, Durham, NC, USA; 2 Stanford School of Medicine, Stanford, CA, USA; 3 University of New South Wales, Sydney, Australia; 4 National Institute for Communicable Disease, Johannesburg, South Africa; 5 Boston University, Boston, MA, USA

## Background

The origins and maturation pathways of broadly neutralizing antibodies (bnAbs) are unknown. Only ~20% of HIV-1-infected subjects develop bnAbs, suggesting their development may require unusual or protracted maturation pathways.

## Methods

Two subjects were followed from the time of HIV-1 infection to >3 years; one developed broad neutralization (CAP206) while the other did not (CH040). Memory B cells were sorted as single antigen-specific cells, Ig heavy and light chain genes were amplified by PCR, and recombinant mAbs produced. Variable heavy chain gene (V_H_) 454 pyrosequencing was performed on 5-10 samples spanning the 3-5 years of infection.

## Results

From CAP206 we isolated 13 Env-reactive mAbs of which 6 (46%) used V_H_1-69; from these we identified three V_H_1-69 clonal lineages. One clonal lineage contained a neutralizing antibody (CAP206-CH12) while the other two lineages had non-neutralizing antibodies (CH64, CH82). All three clonal lineages were mutated (range 5.2-11.8% V_H_ mutation), and members of these lineages could be detected by 454 sequencing as early as one month after infection, with persistence as late as 57 months after infection. In contrast, an autologous neutralizing antibody clonal lineage from CH040 was found only over a one month period, and was not detected in three additional samples over 48 months. Of 18 additional CH040 Env clonal lineages, lineage members from 9 were found only at a single time point, 8 lineages had members found over two time points, and only 1/18 lineages were detected spanning 48 months.

## Conclusion

Multiple Env-reactive antibody clonal lineages persisted for up to 5 years in broad neutralizer CAP206 while the autologous neutralizing antibody clonal lineage and other Env-reactive lineages did not persist in non-neutralizer CH040. These data raise the hypothesis that a high degree of clonal persistence was required for the development of broad neutralization, and imply a predisposition for this trait in broad neutralizers.

